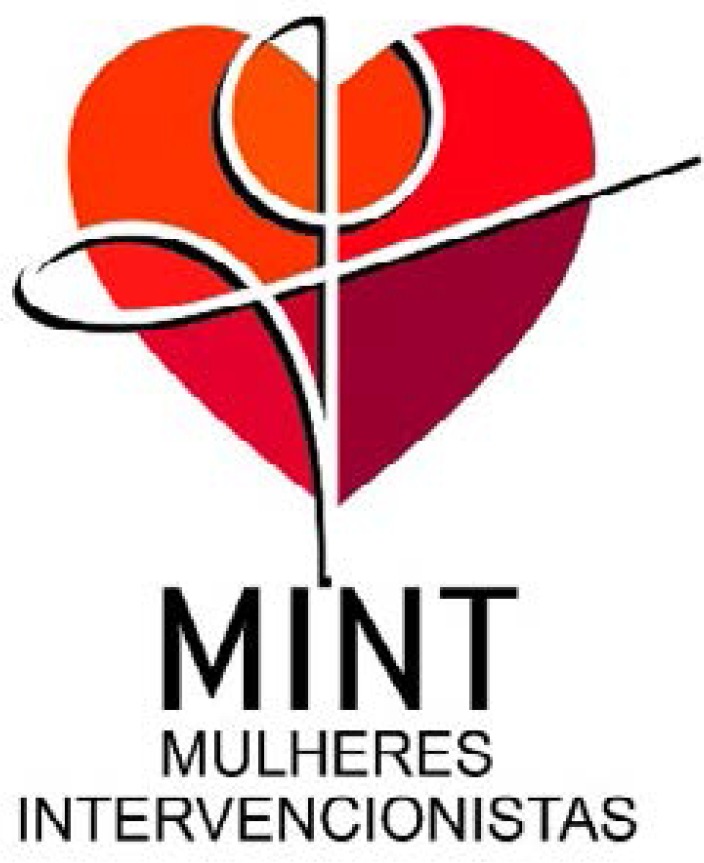# Gender Equity in Healthcare: An Issue of Justice or Need?

**DOI:** 10.5935/abc.20190168

**Published:** 2019-08

**Authors:** Viviana Guzzo Lemke

**Affiliations:** 1 Sociedade Brasileira de Hemodinâmica e Cardiologia Intervencionista - SBHCI, São Paulo, SP - Brazil; 2 Grupo MINT - Mulheres Intervencionistas, Curitiba, PR - Brazil

**Keywords:** Cardiologists, Women, Medicine/trends, Leadership, Gender Identity, Interventionals

With great interest on the topic, we read the article “The Profile of the Brazilian Cardiologist - A Sample of the Members of the Brazilian Society of Cardiology”, by Faganello et al.,^1^ where the professional and personal characteristics of Brazilian cardiologists are reported. The significant gender differences were highlighted in the mini-editorial “Profile of Brazilian Cardiologists: A look on Female Leadership in Cardiology and Stress - Challenges for the Next Decade” by Mesquita et al.,^2^ where peculiarities such as payment and the small number of women in Cardiology are analyzed according to an intriguing point of view.

These articles resonate with the “Women’s Letter” by Oliveira et al.,^3^ a document based on current objectives, which require long-term efforts and structural changes in the medical culture, especially regarding the participation of women in executive positions in medical specialty societies and healthcare-related government bodies.

The important study “Medical demographics in Brazil 2018” by Scheffer et al.,^4^ reports a reality which is already known by cardiologists: despite the fact that women currently represent the majority of students at Medical schools, indicating that doctors up to 34 years of age are mostly women, 70% of Cardiologists are men. This reality further contributes for the small number of women choosing Interventional Cardiology as their specialty.

Acknowledging the need for a greater and more effective participation of women in Medicine and Science as a whole, the Brazilian Society of Hemodynamics and Interventional Cardiology has created the so called “Mulheres INTervencionistas - MINT (Women Interventionists), whose objective is to pursue gender equality at a professional and patient level, encouraging female doctors to choose Interventional Cardiology as their specialty, thus helping improve the odds to have equal career opportunities as men, in addition to increasing the awareness of the interventional and research community about gender-related disparities in the diagnosis and treatment of patients with cardiovascular diseases, supporting the routine participation of women in clinical trials to guarantee women are present in all aspects of scientific literature, be it in clinical trials, guidelines or regulatory processes.

Finally, going back to the remark made by the mini-editorial, sexism cannot bel et aside in the analysis as one for the factors that discourage women to take up medical careers. Struggling for equal conditions and payment must be more than an objective, since, as reported in the important Lancet editorial in February 2019, “Feminism is for everybody”, gender equality is not only a matter of justice and rights, it is essential to produce better research and provide better patient care. It is the duty of medical societies to head this change of paradigma for opportunities to be akin to all, adding forces so that the well known female characteristic, caring for others, may benefit all of our patients.

## Figures and Tables

**Figure:**